# Single Photon Counting UV Solar-Blind Detectors Using Silicon and III-Nitride Materials

**DOI:** 10.3390/s16060927

**Published:** 2016-06-21

**Authors:** Shouleh Nikzad, Michael Hoenk, April D. Jewell, John J. Hennessy, Alexander G. Carver, Todd J. Jones, Timothy M. Goodsall, Erika T. Hamden, Puneet Suvarna, J. Bulmer, F. Shahedipour-Sandvik, Edoardo Charbon, Preethi Padmanabhan, Bruce Hancock, L. Douglas Bell

**Affiliations:** 1 Jet Propulsion Laboratory, California Institute of Technology, Pasadena, CA 91109, USA; michael.hoenk@jpl.nasa.gov (M.H.); April.D.Jewell@jpl.nasa.gov (A.D.J.); John.J.Hennessy@jpl.nasa.gov (J.J.H.); Alexander.G.Carver@jpl.nasa.gov (A.G.C.); todd.jones@jpl.nasa.gov (T.J.J.); timothy.goodsall@jpl.nasa.gov (T.M.G.); bruce.hancock@jpl.nasa.gov (B.H.); lloyddoug.bell@jpl.nasa.gov (L.D.B.); 2 Department of Physics, Mathematics and Astronomy, California Institute of Technology, Pasadena, CA 91125, USA; hamden@caltech.edu; 3 College of Nanoscale Science and Engineering, SUNY Polytechnic Institute, Albany, NY 12203, USA; psuvarna@albany.edu (P.S.); jbulmer@albany.edu (J.B.); sshahedipour-sandvik@albany.edu (F.S.-S.); 4 Department of Microelectronics, Delft University of Technology, Delft, The Netherlands; e.charbon@tudelft.nl (E.C.); PreethiPadmanabhan@student.tudelft.nl (P.P.)

**Keywords:** ultraviolet, quantum efficiency, MBE, ALD, EMCCD, APD, ROIC, Avalanche, visible rejection, MOCVD, GaN

## Abstract

Ultraviolet (UV) studies in astronomy, cosmology, planetary studies, biological and medical applications often require precision detection of faint objects and in many cases require photon-counting detection. We present an overview of two approaches for achieving photon counting in the UV. The first approach involves UV enhancement of photon-counting silicon detectors, including electron multiplying charge-coupled devices and avalanche photodiodes. The approach used here employs molecular beam epitaxy for delta doping and superlattice doping for surface passivation and high UV quantum efficiency. Additional UV enhancements include antireflection (AR) and solar-blind UV bandpass coatings prepared by atomic layer deposition. Quantum efficiency (QE) measurements show QE > 50% in the 100–300 nm range for detectors with simple AR coatings, and QE ≅ 80% at ~206 nm has been shown when more complex AR coatings are used. The second approach is based on avalanche photodiodes in III-nitride materials with high QE and intrinsic solar blindness.

## 1. Introduction

The ultraviolet (UV) spectral range is populated with atomic and molecular lines that are highly relevant for studying planetary bodies, including solar system planets, exoplanets, comets and asteroids as well as stars, supernovae, black holes, galaxies, and the cosmos. In recent years, Hubble Space Telescope (HST) [1,2,3,4], Galaxy Evolution Explorer (GALEX) [5,6], Rosetta [7,8,9,10,11], and the Cassini mission [12] have shown exciting and intriguing results, hinting at and sometimes leading to new discoveries. In depth studies of these phenomena will require further observations with more powerful UV instruments. Discoveries of plumes on Europa, oceans on Enceladus, and theories of intergalactic medium (IGM) and circumgalactic medium (CGM) have opened new scientific questions and windows of study that require improved UV detection capabilities. Using UV spectroscopy and imaging spectrometry, thin atmospheres and surface composition of primitive bodies can be examined. UV emission lines and bands from H, C, O, N, S, OH and CO; UV absorption lines by CO_2_, H_2_O, NH_3_, N_2_; and UV surface reflectance spectra are all essential for the detection of ice, iron oxides, organics, and other compounds on planetary bodies. All of these are used as diagnostic tools for understanding the nature and habitability of these bodies. In addition to space applications, UV is used in defense applications, cancer detection, bacterial detection, machine vision, wafer inspection, lithography, and electrical safety inspection. 

Photon counting is a key capability enabling faint object detection with NASA’s UV instruments. In addition, visible-blindness, or more generally speaking, out-of-band rejection, is often required to detect UV signals in the presence of a significant visible background. UV instruments have traditionally addressed these requirements using image-tube technologies, such as photomultiplier tubes (PMTs) and microchannel plates (MCPs).

Silicon-based imaging detectors with single photon counting capability in the UV represents a significant leap forward in detector manufacturability, accessibility, and reliability. The enormous investment by industry and defense organizations has led to the development of large-format, high-resolution silicon imaging arrays. Relatively recent advances in solid-state imaging technology have produced detector architectures with high efficiency and built-in gain [13,14,15]. Challenges are posed, however, in producing silicon detectors with high UV sensitivity, stability with respect to illumination history, photon counting ability, and out-of-band rejection. Nanoscale surface and interface engineering technologies for surface passivation, together with progress in large-scale production, address these challenges and are leading to solid-state imagers being highly competitive and even superior in performance and cost to replace image-tube technologies in UV instrumentation [16,17,18].

In this paper we discuss two different approaches to solid-state, photon-counting, UV imaging and detection. In the first approach, we employ back-illumination processes developed in our laboratory, including Molecular Beam Epitaxy (MBE)-based superlattice and delta doping as well as Atomic Layer Deposition (ALD)-based custom antireflection (AR) coatings on electron multiplying charge-coupled devices (EMCCDs) and integrated filters on avalanche photodiodes (APDs). Our recent results show record high UV quantum efficiency (QE) of 60%–80%. Integrated metal dielectric filters (MDF) are used for visible rejection. We have measured dark current and clock-induced charge at low enough levels to make these detectors attractive and competitive for photon counting applications. In the second approach, we use gallium nitride and its alloys in a hybrid APD design. Gallium nitride and its alloy gallium aluminum nitride are wide bandgap materials that, depending on the fraction of aluminum in the alloy, span a wide range of direct bandgaps with tailorable out of band cutoff from 3.4 eV to 6.2 eV. We have achieved 50% QE in GaN and AlGaN APDs. Due to lack of native substrates, III-N’s suffer from defects and leakage. ALD’s nanoscale precision and conformal coating capability were used for sidewall passivation against leakage, resulting in consistent improvement over detectors coated using Plasma Enhanced Chemical Vapor Deposition (PECVD). Both approaches have resulted in better than 10^4^ out-of-band rejection, which is at the same level as the traditional image tube-based UV detectors. 

We present results for both of these promising approaches as part of our overall focused effort in developing technologies and instrumentation for challenging UV science where single photon counting is required. We discuss concepts, designs, fabrication and processing, and characterization techniques.

## 2. Materials and Methods: Silicon and Gallium Nitride/Gallium Aluminum Nitride Detector Designs with Avalanche Gain

### 2.1. Single Photon Counting in the UV with Silicon 

#### 2.1.1. Silicon Detectors with Gain

Avalanche multiplication has been used in various silicon detector architectures to achieve signal gain, including APDs, APD arrays, single photon avalanche photodiode (SPAD) arrays, and EMCCDs [15]. It is now possible to achieve single photon counting in silicon arrays rivaling that achieved with high-voltage photocathode-based imaging technologies, provided that the noise levels are still kept low. The focus of this work is the combination of delta doping and custom AR coatings to achieve high and stable QE for single photon counting imaging detectors, particularly in the UV. Two types of silicon detectors with avalanche gain were used. A brief description of each detector follows. 

##### Electron Multiplying CCDs

**Figure 1:**
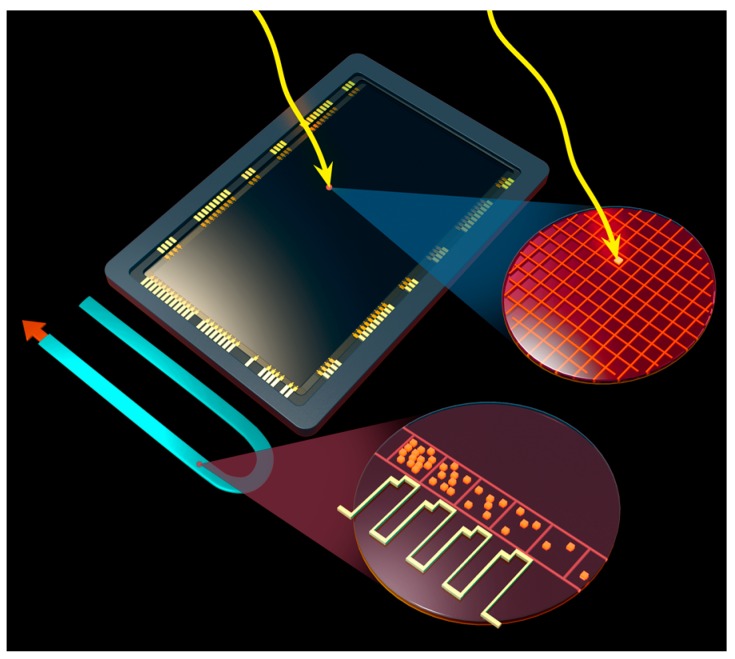
Artist’s concept of the avalanche gain process in an Electron Multiplying Charge Coupled Device (EMCCD). The EMCCD architecture is the same as a regular two-phase CCD but with an added serial register stage where additional voltage is applied at each transfer that induces probability of avalanche. Photons impinge on CCD pixels resulting in the generation of photoelectrons as depicted in the CCD and the blow up circle on top right. The photoelectron is transferred through normal CCD charge transfer process until it reaches the special gain register of the EMCCD. In this gain register, avalanche multiplication is induced by applying higher than normal clock voltages (~40 V) as shown in the figure blow up. The CCD shown in the figure is an artist’s recreation of e2v‘s (Chelmsford, UK) CCD201.

EMCCDs leverage the advantages of the mature CCD technology while enabling single photon detection. Charge amplification is achieved in EMCCDs by appending a gain serial shift register to the serial register of a conventional CCD. At each stage of this gain serial shift register, a higher gate voltage (~40 V) in the second serial clock phase causes an impact ionization effect (Figure 1). The impact ionization effect produces a small gain in each charge transfer, enabling a cumulative (mean) charge gain of more than one thousand [13,14]. 

Charge gain in EMCCDs mitigates output read noise. Low light level sensitivity in EMCCDs is limited by low-level sources of spurious signal, such as dark current and “clock induced charge” (CIC). Clock induced charge, which is generated in the detector pixels during the readout process, is commonly attributed to electric field-induced hole transport between the detector surface and column stops. The generation rates are tied to bias voltages and surface inversion during clocking. Although relatively insignificant in most CCDs, CIC can be a source of spurious signal in EMCCDs [13,14,19]. As demonstrated by Hamden *et al.*, these remaining spurious signal sources are manageable by conventional techniques and have recently been measured for the Faint Intergalactic Red-shifted Emission Balloon (FIREBall-2) experiment [20]. Cooling (<−120 °C) and reducing exposure from array saturating sources can reduce dark current to levels well below the typically reported 1 e^−^-pixel^−1^-hr^−1^ [21], leaving CIC as the limiting noise source. There are simple yet powerful techniques to reduce the effects of the CIC such as optimal wave shaping filters applied to the CCD clocks [22,23]. Furthermore, because CIC results from charge transfer, its impact increases proportionately with frame rate. Many scientific applications do not require a high frame rate, and therefore can be read out only as often as necessary, for example, to minimize the effects of cosmic rays (~1000 s). 

##### Avalanche Photodiodes

Unlike conventional photodiodes, APDs are designed to sustain high bias voltages without breaking down. In large area linear-mode APDs, high electric fields in the region of the p-n junction can generate multiplication gains as high as 1000×. The UV sensitivity and response time of standard linear-mode APDs are limited by diffusion and recombination in the undepleted silicon near the surface. In a collaboration between Radiation Monitoring Devices, Inc. (RMD, Watertown, MA, USA), the California Institute of Technology (Caltech) and the Jet Propulsion Laboratory (JPL), we have demonstrated superlattice-doped APDs in which the depletion region can approach the surface, thereby enabling high UV sensitivity and fast response. Superlattice doping in these devices has also enabled the development of visible-blind, UV band pass filters, with high QE in the deep ultraviolet, and excellent out-of-band rejection.

#### 2.1.2. Silicon Passivation

The concept of backside illumination (BSI) was created in the 1970s to reduce the losses in the front-side device circuitry, effectively increasing the QE and expanding the spectral response into the UV range [24,25,26]. Today, BSI detectors have become prevalent even in commercial applications; however, many of the processes needed for BSI were initially developed for scientific detectors, with HST’s Wide Field/Planetary Camera-1 (WF/PC-1) leading the way as the first implementation of the idea in space. As was quickly discovered, these early BSI silicon detectors had poor due UV efficiency due to the shallow absorption depth of UV photons and limitations of then-available technologies for thinning and surface passivation. To circumvent these limitations and enable the detection of UV photons in HST’s WF/PC-1 instrument, backside-thinned CCDs were coated with phosphors such as coronene or lumogen that convert UV photons into visible photons that could be detected more efficiently. Unfortunately, the performance of WF/PC-1 CCDs was severely compromised by instabilities known as QE hysteresis (QEH). QEH in BSI detectors is characterized by low and unstable sensitivity, especially at the blue end of spectrum. QEH is a consequence of poor surface passivation, as unpassivated surface defects trap photogenerated electrons and holes, resulting in time-variable surface charge and surface depletion depth. Despite another two decades of development, state-of-the-art ion-implanted CCDs currently flying in Hubble’s Wide Field Camera 3 instrument still exhibit QEH at a level of several percent, which is a testament to the importance and difficulty of surface passivation [27].

The surface of thinned, BSI detectors comprises high purity silicon, which is extremely sensitive to charge in surface oxides and at interfaces. Charge transfer from silicon to interface traps causes the Si-SiO_2_ interface to acquire a net charge. At thermal equilibrium, p-type silicon surfaces acquire a net positive charge due to trapping of majority carriers. The physical location of traps in the oxide plays an important role in the dynamics of the illuminated surface. Defects and contaminants create a fixed charge in the oxide, while traps at the Si-SiO_2_ interface acquire a variable charge depending on the near-surface Fermi level and the time-dependent illumination of the surface. Traps located within ~1 nm of the silicon surface interact with mobile charge in the underlying silicon, and are therefore capable of changing charge state in response to transient changes in the surface potential. Traps located deeper in the oxide change state much more slowly. 

The net charge trapped in oxide and interface states generates an electric field that depletes the nearby silicon. From a device physics standpoint, interface traps “pin” the surface Fermi level in the middle of the bandgap. In general, the silicon surface is transformed into a “surface dead layer” that renders the detector insensitive to light that is absorbed near the surface. In order to achieve high efficiency and stable response, it is therefore necessary to combine thinning of BSI detectors with a surface passivation technology that reduces or eliminates the surface dead layer while at the same time suppressing the surface-generated dark current associated with a high density of interface traps. 

Since the first BSI CCDs were demonstrated in 1974 [24], many methods have been developed for back surface passivation. These can be roughly divided into backside charging and back surface doping technologies, which are distinguished by the location and distribution of charge in the detector and its oxide. Backside charging methods stabilize the detector by introducing negative charge into the oxide. Provided that the density of interface traps is not too high, the negatively charged passivation layer will bias the surface into accumulation. Backside doping methods take the opposite approach by introducing an impurity profile that stabilizes the surface and creates an internal bias in the detector.

The advantage of surface doping lies in the potentially greater stability that can be achieved. In particular, surface doping promises greater resistance to radiation-induced traps. In p-type surfaces, holes are trapped in interface states, depleting the surface of holes and forming a depletion layer with a negative net charge density. The surface is therefore characterized by a charge dipole, bounded on one side by positive charge in the surface/oxide and on the other by the negatively charged depletion layer. To a first approximation, the depth of the depletion layer is given by the ratio of surface charge density to silicon dopant density. Thus, variations in the surface charge density are reflected by variations in the depletion layer depth. Within the depletion layer, photogenerated electrons experience a force directed toward the surface where they can be lost to trapping and recombination. Back surface doping methods seek to create a near-surface doping profile that simultaneously shrinks the surface depletion layer and creates a strong, built-in electric field. Controlling the doping profile can stabilize the detector against variations in surface charge density. Ideally, surface doping achieves a high near-surface dopant concentration with a strong gradient, thereby creating a surface that is insensitive to charge trapped at the surface. Surface doping is potentially more stable than back surface charging in challenging environments, including high radiation environments in space.

Unlike conventional “3D” doping methods, in which dopants are randomly distributed in the silicon lattice, 2D doping (also known as delta-doping) incorporates dopant atoms in highly ordered, self-organized two-dimensional layers. For p-type doping with boron, at concentrations >3 × 10^20^ B/cm^3^ the conventional (3D) approach suffers from poor quality crystals, in which dopant atoms are only partially activated due to clustering and incorporation into interstitial sites. In contrast, 2D doping is marked by high crystal quality with nearly 100% electrical activation at concentrations exceeding the 3D doping limit by an order of magnitude. The growth of 2D-doped silicon is based on self-organized surface phases that form during sub-monolayer deposition of dopant atoms on silicon. Once formed, these self-organized surface phases are stabilized by covalent bonds in the silicon lattice. In our work, we have routinely used 2D sheet densities of 2 × 10^14^ B/cm^2^; however, 2D-doped surfaces with single-layer sheet densities as high as 3 × 10^14^ B/cm^2^ have been demonstrated.

**Figure 2:**
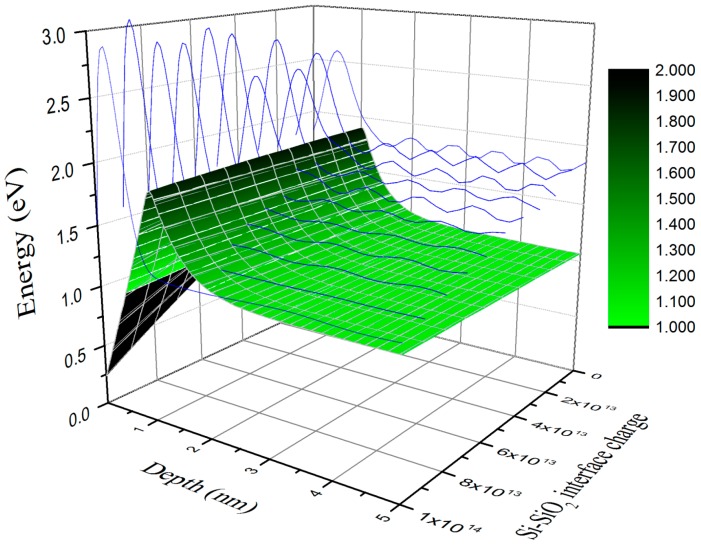
Three-dimensional plot of conduction band edge *vs*. depth and silicon-silicon oxide interface charge density for band structure engineered, 2D-doped BSI silicon array (shown for a delta-doped device). The figure shows that the placement of a high density of boron in a single atomic sheet creates a delta function change at the edge of the conduction band (green plot). The electron wave functions (shown in blue) are unconfined, illustrating that the placement of a delta layer within 1 nm of the surface reduces the probability of photoelectron trapping. The figure illustrates the stability of the 2D-doped surface against variable surface charge, which is associated with interface traps and radiation damaged surfaces.

An additional major challenge in fabricating BSI detectors is that standard surface passivation processes require high temperatures that would damage the detector. Low-temperature surface passivation technologies have been developed, but many—such as ion implantation and laser anneal—still suffer from lower QE or QE instability as a function of environment or illumination history. JPL-invented low-temperature MBE has been developed as a method of passivating BSI detectors [28,29]. This approach enables control over the surface doping profile with nearly atomic-scale precision (Figure 2). The precision control of surface band structure engineering and interface engineering afforded by 2D doping by MBE results in high QE without any QEH [16,17,30]. The first time JPL passivated a CCD using MBE, the passivation layer comprised 5 nm of silicon doped at 3 × 10^20^ B/cm^−3^, *i.e*., 3-D doping, plus a sacrificial 1-nm un-doped silicon cap layer to form the surface oxide [29]. In all subsequent devices, JPL has used 2D doping for surface passivation [17,18,28,30,31,32,33,34,35,36,37,38]. During MBE growth, a thin layer of un-doped silicon is grown on the substrate to form an atomically clean, uniform silicon surface. The silicon flux is interrupted by closing a shutter, and boron is deposited on the atomically clean silicon surface, where it spontaneously forms a self-organized surface phase, as described above. Once the surface coverage reaches a pre-defined level, the boron shutter is closed and the silicon shutter is opened in order to grow a thin layer of crystalline silicon on the boron-doped surface. This process is termed delta doping, because the resulting vertical dopant profile resembles the mathematical delta function. Occasionally we will refer to “superlattice-doping” in which more than one delta layer is included in the MBE structure.

#### 2.1.3. Atomic Layer Deposition for Antireflection Coatings & Detector-Integrated Visible-Rejection Filters

Many of the concepts of AR coatings and visible rejection filters for the far UV spectral range have been developed over the years, but could not be implemented on live devices with accuracy or fidelity [35]. ALD is a technique that is a close spinoff of chemical vapor deposition (CVD). ALD films are created a single atomic layer at a time through a series of self-limiting chemical reactions with the substrate surface; different reactants designed for layer-by-layer growth are introduced sequentially, followed by purging (e.g., N_2_, Ar) to remove reaction byproducts after each layer is formed. This chemical-reaction-driven process allows for the precise control of stoichiometry, thickness, and uniformity, while producing highly dense and pinhole free films with sharp and well-defined interfaces. These characteristics make ALD ideal for preparing optical coatings based on single and multilayer films, and we have employed this technique to develop highly effective AR coatings in the challenging far UV spectral range [34,37,39,40].

There are cases in which it is highly desirable to extend a UV detector’s sensitivity to visible and infrared wavelengths. Silicon detectors are ideal for such cases, for example spectroscopic applications where broadband response is needed. However, there are also cases in which a detector’s sensitivity to visible photons introduces an undesirable signal that interferes with UV measurement. This type of out-of-band background, also known as “red leak”, is particularly problematic in environments with weak UV signals masked by presence of a strong visible signal. For these applications, some degree of visible-blindness or out-of-band rejection is required. 

To achieve visible rejection, we turn to metal dielectric filters (MDFs). As stand-alone filters, these Fabry-Perot structures, which are also referred to as photonic bandgaps, have been used in the past as bandpass filters for spectral ranges from the UV to the infrared [41,42,43,44]. Briefly, the metal layers are separated by transparent dielectric spacer layers. The layered structure is designed to resonantly transmit light within a specific wavelength band, while out-of-band light is strongly reflected by the metal layers. The in-band light suffers absorption losses in the metal layer; therefore, it is necessary to choose metals with a large absorption coefficient to index of refraction (k/n) ratio in the band of interest. In the UV the primary choice is aluminum due to its high plasma frequency and relative lack of significant interband transitions in this spectral range [45,46].

**Figure 3:**
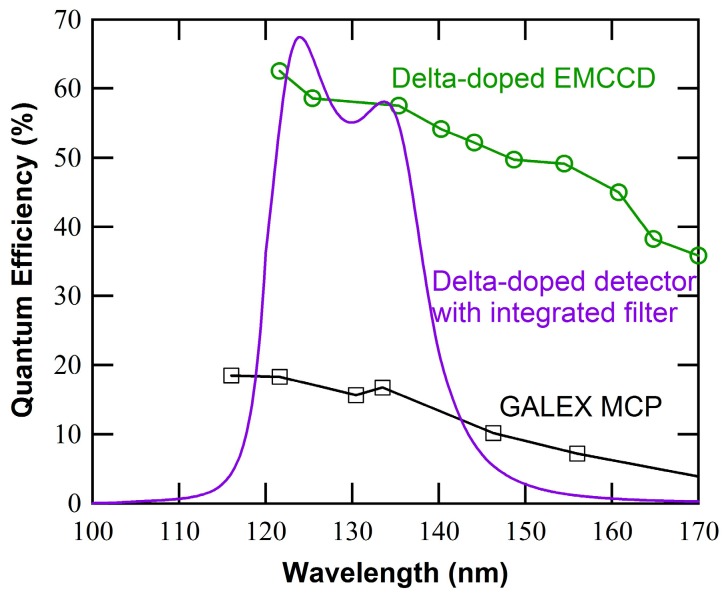
Model performance of a 2D-doped detector with an integrated visible-blind bandpass filter for far UV wavelengths. The multilayer MDF was designed to provide high in-band QE, and high out-of-band rejection (>10^4^). As designed the MDF includes layers of MgF_2_ (20 nm thick), Al (26 nm), MgF_2_ (18 nm), Al (20 nm), and MgF_2_ (11 nm), starting from the silicon interface up to the air interface.

Designs of this type have been extended to Si substrates for potential use as a filter directly integrated on a photodetector, making it possible to at once optimize the in-band sensitivity together with the out-of-band rejection [47]. The complexity of the filter design influences several performance metrics, including peak transmission percentage, out-of-band rejection ratio, passband width, and target wavelength. Depending on the target wavelengths these filter designs may be based variety of dielectric materials, including MgF_2_ as illustrated in Figure 3. ALD remains a critical aspect of this work, especially for complex multilayer stacks, which requires accurate thickness control, layer uniformity, and precise control of the interface chemistry between the metal and dielectric layers.

#### 2.1.4. Large-Scale, High Throughput Affordable Production of High Efficiency Single Photon Counting Silicon Imagers for Missions and Commercial Applications

With the need for high throughput processing of scientific detector arrays as well as high throughput processing for commercial use apparent, a scaled up version of our post fabrication end-to end processing was developed over the last few years. The scaling up process began with upgrading the MBE capability by design, procurement, and commissioning of a multiple wafer batch mode, wafer-scale MBE machine. All the steps of the process can be performed at wafer-scale or die level and can be customized based on project needs. 

**Figure 4:**
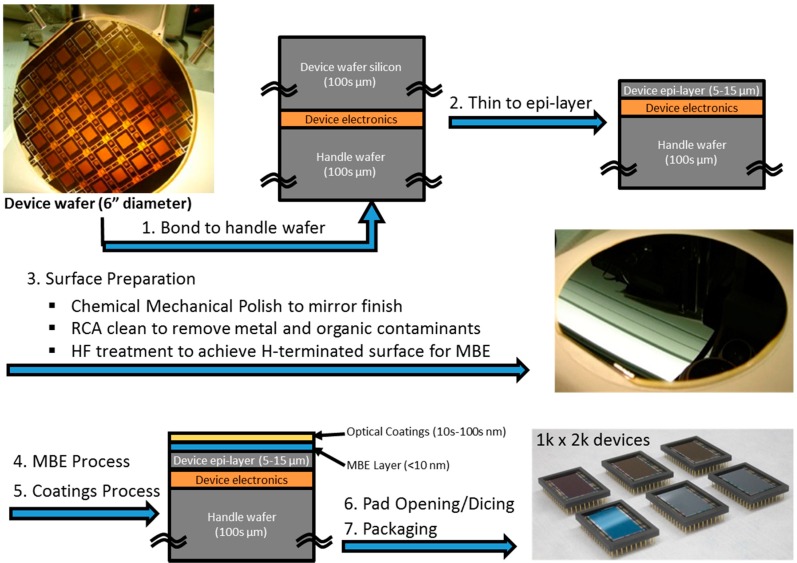
Process flow for end-to-end post fabrication processing along with schematic diagram of a superlattice-doped or delta-doped arrays (summarized as MBE layers), and the photographs of bare and AR-coated BSI silicon detector arrays. The coated arrays can be discerned from the reflective hue seen in photograph.

Figure 4 shows JPL’s end-to-end post-fabrication processing as applied to a “generic” device type. This example is illustrative of a process flow that can be customized and adapted according to the requirements of a wide variety of silicon detectors. The process begins with fully-fabricated detector arrays, complete with final metallization. The VLSI (Very-Large-Scale Integration)-fabricated circuitry and pixel structure are protected by direct oxide-oxide bonding of the device wafer’s front surface to a blank silicon wafer that has an identical diameter and is a few hundred microns thick. The blank wafer also serves as a mechanical support once the device wafer is thinned down to the epitaxial silicon layer thickness, *i.e*., a few micrometers to tens of micrometers thick. The oxide-oxide bond must be devoid of any epoxy or organics, in order for the bond to survive subsequent high temperature processing steps, including MBE and ALD. Following direct wafer bonding, the bulk of the detector substrate is removed by grinding and chemical mechanical polishing, which reduces the device wafer thickness from hundreds to ~50 µm. The remainder of the substrate is removed by a selective thinning process, using an isotropic chemical etchant that removes the bulk P+ Si at a rate of >100:1 compared to the P-epilayer. This dopant-sensitive selectivity enables the use of the epilayer as an etch stop. Final polishing produces a smooth mirror finish surface that will be subjected to a simple series of cleaning steps to prepare the surface for epitaxial growth.

The backside-thinned detector wafer is then passivated by MBE growth of 2D-doped Si layers, comprising single or multilayer stacks of delta-doped Si as described in Section 2.1.2. MBE growth provides the precise band structure engineering of the Si surface to allow detection of higher energy photons (100 nm < λ < 400 nm) that are absorbed in shallow depths (4–10 nm) with minimal to no loss of photoelectrons [16,18].

After passivation, wafers are moved into the ALD machine. A thin oxide layer is grown to protect the 2D-doped surface. AR coatings or filters can be applied to the entire wafer at this time; alternatively, the wafer can be diced and coatings applied to individual or multiple die. As described in Section 2.1.3, our optical coatings are typically prepared by ALD, which enables atomic-scale control of structure and composition, including multilayer coatings with embedded metal films for bandpass coatings with high in-band QE and excellent out-of-band rejection [36].

### 2.2. Single Photon Counting in the UV with III-Nitride APDs 

The III-Nitride material family can be alloyed to span the group of direct bandgap semiconductors spanning in bandgap range from 3.4 to 6.2 eV. Due to their wide bandgaps, they are naturally insensitive to visible photons, the cutoff wavelength can be tailored and the resulting devices can be operated at higher temperature. Most III-Nitride materials are grown on sapphire or silicon substrates. This is because there are no native substrates except for bulk substrates produced by hydride vapor phase epitaxy (HVPE) which are small and costly. The growth on mismatched substrates leads to defects and consequently leakage when devices are processed.

Our approach in developing III-Nitride-based single photon counting detectors in the UV has been to use novel growth techniques in order to start from the optimized material quality; to use design and processing techniques for reduction of leakage and improvement of efficiency; and finally to design readouts that can address the need for the high avalanche voltage and the quenching of breakdown possibility. Here we discuss some special aspects of growth, processing and readout and in the results section we show quantum efficiency, out-of-band rejection, tailorability, and gain of APDs formed of GaN and AlGaN material. 

#### 2.2.1. Brief Description of the Special Features of III-Nitride Materials Growth

For the work described here, the team from the College of Nanoscale Science and Engineering (CNSE) at State University of New York (SUNY)-Polytechnic performed all device growth development and contributed to device design, fabrication and processing. Growths were performed using Metal Organic Chemical Vapor Deposition (MOCVD). Novel features of the growth are briefly described here in experiments using two different substrates. 

**Figure 5:**
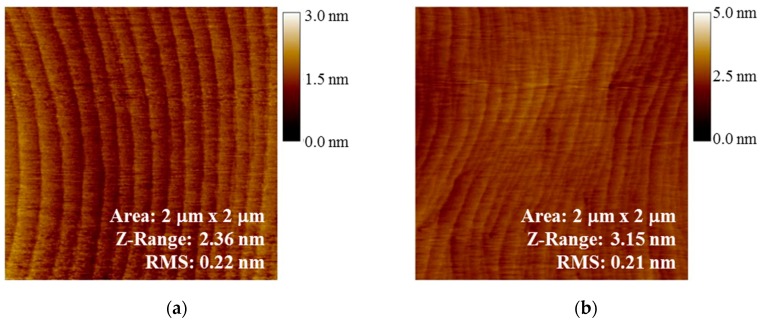
AFM images of MOCVD GaN grown on (**a**) HVPE GaN and (**b**) GaN template on sapphire showing high quality materials growth on the native substrate.

Here we have used HVPE GaN substrates for the growth that creates smooth and high quality material. GaN p-i-n APDs were grown on HVPE bulk substrate using MOCVD. The high quality of the material grown on bulk substrates is evident from the smooth atomic force microscopy (AFM) image of the GaN surface showing uniform parallel step edges, in comparison to the surface on sapphire that shows step terminations and defects (Figure 5).

For some applications, including those that require solar blind operation, AlGaN APDs with [Al] > 40% need to be developed; this brings with it many more challenges in addition to the problems already discussed for GaN APDs. Conventional growth of Al_x_Ga_1-x_N with x > 0.4 (required for cut-off wavelengths below 280 nm) is problematic, producing highly defective films due to gas phase reactions and low surface adatom mobility. We have recently developed a new technique for growth of high quality, high Al composition AlGaN films, where the Al and Ga precursors and ammonia are sequentially pulsed during the MOCVD film growth. The process has some similarities to the growth protocol used for ALD, and improves the material for similar reasons: growth species have time to saturate the surface and move to stable sites before the next species is introduced. Pulsed growth also addresses another MOCVD limitation: in some cases, MOCVD precursors partially react in the gas phase before reaching the growth surface. By sequentially introducing the precursors and purging the system between pulses, the precursors cannot interact in gas phase. Using these new pulsed growth methods, we have maintained material quality while increasing doping levels by about an order of magnitude. This gives us very high quality films, with X-ray diffraction from the 0002 crystal orientation yielding peak widths (FWHM) of less than 100 arcsec.

This is especially important for higher doping levels in AlGaN, since doping becomes more difficult as the Al fraction increases, eventually resulting in a decrease in material quality. Due to the low activation of dopants in AlGaN (~1%), much more dopant must be incorporated in AlGaN than in GaN to achieve the same carrier concentration, leading to difficulty in maintaining the crystalline quality of the AlGaN. Pulsed growth allows higher carrier concentrations while maintaining sufficient material quality.

**Figure 6:**
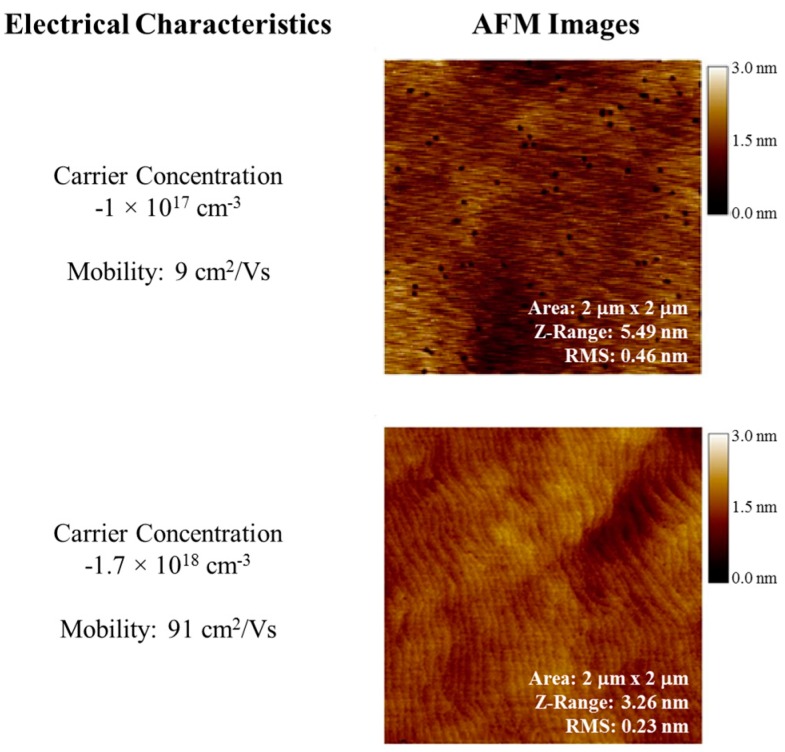
Pulsed growth in MOCVD shows marked improvement in the quality of AlGaN films as shown in the AFM images. The higher quality of both films is the result of the pulsed growth. Introduction of Si during the pulsed sequence is shown to greatly impact defect formation and density as well as carrier concentration. Si dopants were introduced during the Al pulse in the AFM image (**top**), whereas introduction of Si during the Ga pulse (**bottom**) increases both the free electron concentration as well as marked improvement in the quality of the AlGaN film.

Growth parameters were varied to get-high quality Si-doped Al_0.6_Ga_0.4_N films, which show a carrier concentration of 2 × 10^18^ cm^−3^ and mobility of 90 cm^2^∙V^−1^∙s^−1^. The crystalline quality and electrical properties achieved by our team are the state of the art for high aluminum percentage AlGaN. Using pulsed growth technique, we have been able to grow crack-free AlGaN films with thickness greater than 500 nm on sapphire substrates. The values of carrier concentration and mobility of the pulsed n-AlGaN films are among the best reported results for high aluminum percentage n-AlGaN on sapphire. Values of electrical characteristics are given in Figure 6.

#### 2.2.2. Processing Features 

**Figure 7:**
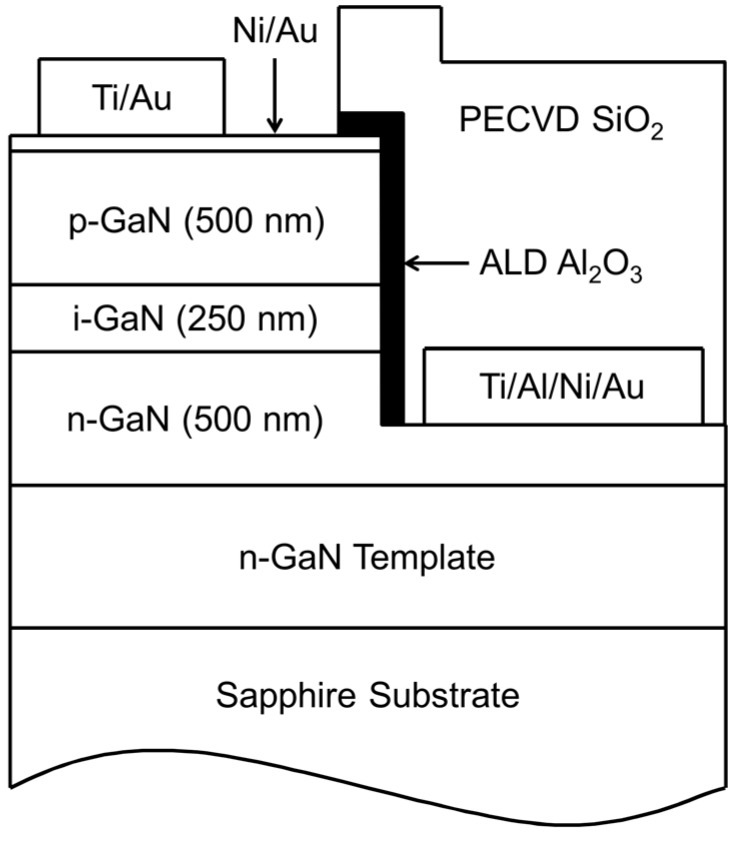
Schematic of device geometry showing the application of the ALD Al_2_O_3_ layers as sidewall passivation and incorporating PECVD SiO_2_ as a contact isolation layer. In devices not receiving the ALD treatment, the mesa sidewalls are passivated by the PECVD film only.

Some of the typical challenges facing APD production are device yield, robustness, and dark current. In particular, sidewall-related defects in GaN APDs have often been observed to contribute to undesirable current components, such as those produced by defect-related microplasmas. SiO_2_ is most commonly used for passivation due to its availability and simplicity of growth; Figure 7 shows an example device structure and one implementation of the sidewall passivation using SiO_2_. SiO_2_ is not the ideal passivation material for AlGaN materials; thus, we are exploring other passivation materials based on ALD processes. ALD is particularly well suited for passivation because the technique results in conformal coatings with monolayer uniformity, even for complex geometries. 

#### 2.2.3. Readout Design and Fabrication for III-Nitride APDs 

The III-Nitride sensor discussed here is a hybrid structure where the detector is processed in III-Nitride material and the readout integrated circuit (ROIC) is designed and fabricated in silicon; the two pieces are hybridized using indium bump bonding. The major part of the effort up to this point has been focused on detector design and fabrication. Recently, in collaboration with the Delft University of Technology (TU Delft), we have begun the work on ROICs. Here we describe some of the major parameters considered for the readout and the particular challenges for this detector. In the results section, simulation plots and preliminary fabrication results are discussed. 

**Figure 8:**
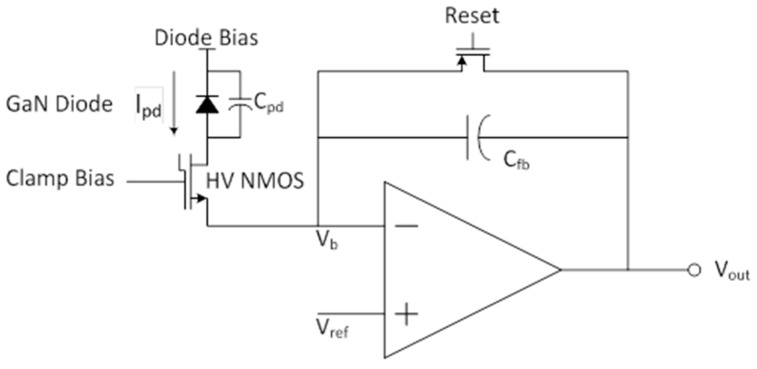
Block diagram of a capacitive transimpedence amplifier basic concept used for the readout of the GaN detector.

Based on the detector characteristics, an equivalent diode circuit model was chosen, consisting of the photodiode current along with its equivalent capacitance. One of the goals was to keep the design as simple as possible while obtaining a suitable match with the detector characteristics and adequate readability at the readout output. These considerations led to a Capacitive TransImpedance Amplifier (CTIA) as a feasible solution, as shown in Figure 8. Initially, the feedback capacitor (C_fb_) is discharged by the reset transistor and the output equals V_ref_. Once the reset is released, incoming photodiode current (I_pd_) passing through the high voltage N-type Metal Oxide Semiconductor (NMOS) transistor is integrated on C_fb_. The amplifier generates whatever output is necessary to keep the input voltage zero as the capacitor charges up. Thus, the output swings negative by a voltage equal to the integrated charge divided by the feedback capacitance.

One challenge with these detectors is that the voltage required for electron multiplication is high, on the order of 60–100 V. Readout circuits must be protected from these high voltages. A convenient solution, available in the chosen Complementary Metal Oxide Semiconductor (CMOS) process, is the use of the high voltage NMOS clamp transistor. The clamp bias is set so that, initially, the clamp transistor is in its ohmic regime. There is very little voltage drop across it and the photodiode sees its full bias. Eventually, however, the amplifier output will saturate. Thereafter, the current charging C_fb_ will begin to raise the voltage of the positive input. Left unchecked, it could reach damaging levels. Instead, with this circuit, as the amplifier input voltage rises the gate-to-source voltage of the clamp transistor is reduced and the transistor begins to shut off. The bias is chosen so that shutoff occurs before the input exceeds a safe voltage. Once the transistor shuts off, the photodiode current (I_pd_) no longer flows through it to charge C_fb_, instead charging the photodiode capacitor (C_pd_) and reducing the diode bias. In the case of avalanche breakdown the diode current will be high and the processes will happen quickly: saturation of the amplifier, shutoff of the clamp transistor, and debiasing of the photodiode. The debiasing of the diode, in turn, will quench the avalanche. A high voltage will remain on the drain of the clamp transistor, but it is designed for this. In order to resume operation, the reset transistor must be operated. This will pull down the source of the clamp transistor, turning it on. I_pd_, no longer high, will sink through the amplifier output, along with the current needed to charge C_pd_ back to its full bias voltage. When the reset is released, the process begins anew.

Our implementation of this concept uses a simple single-ended common-source PMOS transistor for the amplifier. An NMOS source follower was added to buffer the output of the feedback amplifier for monitoring, and another to allow monitoring the amplifier input voltage. This should be useful for observing the increase in voltage when saturation occurs, and should be especially valuable in detecting avalanche breakdown.

## 3. Results

This section describes the results of superlattice-doped EMCCDs and APDs—with AR coatings and visible-blind filters followed by the results of the III-Nitride APDs.

### 3.1. Silicon Detectors

#### 3.1.1. Quantum Efficiency in the Ultraviolet Spectral Range

**Figure 9:**
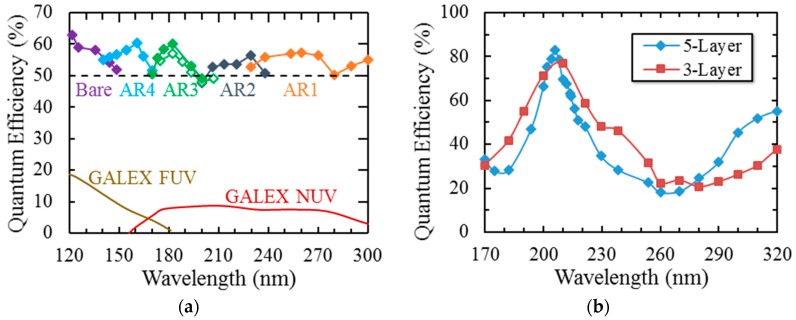
(**a**) QE data of delta-doped conventional (closed diamonds) and EMCCDs (open diamonds) enhanced with single layer AR coatings [34]. (**b**) QE data from two superlattice-doped CCD201s (e2v’s 1k × 2k EMCCD) optimized for the 200–220 nm wavelength range; the device designs included a three-layer AR coating (red squares) and a five-layer AR coating (blue diamonds).

Quantum efficiency results of 2-D growth as single layer (delta doping) and multilayer (superlattice doping) are well documented showing 100% internal QE from soft X-ray to near infrared in various silicon detector architecture and designs [16,28,31,34,48,49]. Addition of AR coatings has shown high external QE tailored for various parts of the spectrum. Here we show two examples of results obtained from back illumination and passivation with 2D growth combined with custom coatings in the FUV and NUV for EMCCDs. Results of this process as manifested in QE are shown in Figure 9 [34,37,40]. Figure 9a shows the QE results of coated delta doped arrays for an imaging spectrometer that covers the challenging part of the spectrum from 100–300 nm. Coatings are simple, single layer designs that result in better than 50% external QE. Figure 9b shows the QE measurements of two superlattice-doped, two-megapixel EMCCDs with two different ALD multilayer AR coatings designed for the atmospheric window of a balloon, aiming to maximize the QE at 205 nm. The higher number of multilayers result in improvement in the QE and narrowing of the peak.

#### 3.1.2. Visible Rejection Using Metal Dielectric Films

**Figure 10:**
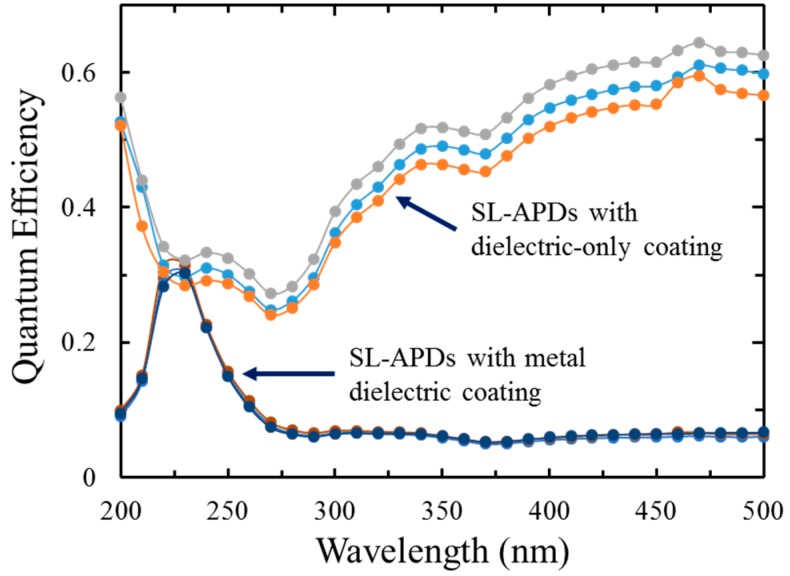
An example of ALD multilayer stacks of metal-dielectrics films, shown in figure is a single metal layer sandwiched between two dielectric layers. The results shown here represent the application of superlattice doping and AR coating technologies to APDs fabricated and characterized by RMD.

The high QE of 2D-doped silicon imagers combined with integrated red-rejection filters is a potentially disruptive sensor technology for applications currently dominated by low QE MCPs. Preliminary results were recently reported based on die-level coating of superlattice-doped APD detectors [16]. Figure 10 shows QE measurements from these superlattice-doped APDs with both MDFs and AR coatings. The MDFs were formed by ALD-grown aluminum oxide and *ex-situ* e-beam evaporated metallic aluminum, while the dielectric-only coatings comprise ALD-grown thermal Al_2_O_3_. A comparison of the dielectric-only coating with the metal-dielectric multilayer stack clearly demonstrates the concept. With a single embedded metal layer, a rejection ratio of near an order of magnitude is achieved. With multiple embedded metal layers, rejection ratios from 10^4^ to 10^7^ are projected. This significant achievement now makes possible UV-sensitive Si detectors with tailorable out-of-band rejection.

This initial demonstration involves an intermediate air exposure of the metallic Al layer prior to ALD Al_2_O_3_ encapsulation which is estimated to result in the formation of 1–2 nm of interfacial Al oxide. For this application at λ > 200 nm, the impact of the oxidation layer is minimal on the final performance, but for applications below 200 nm the oxide absorption losses will begin to limit the predicted peak transmission. For these shorter wavelength applications (which utilize ALD metal fluoride dielectric layers) we are exploring vacuum transfer approaches to limit environmental exposure, as well as methods incorporating atomic layer etching to chemically remove the Al surface oxide immediately prior to ALD encapsulation.

### 3.2. III-Nitride APDs

**Figure 11:**
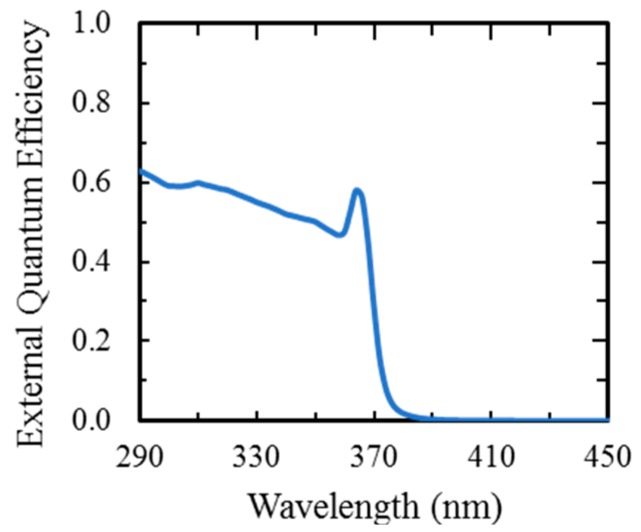
External QE as a function of photon wavelength for an Al_2_O_3_ –passivated GaN p-i-n APD with zero applied voltage. With no applied voltage, there is unity internal gain.

Photodiodes and photodiode arrays were processed from the III-Nitride material and QE was measured in comparison with a calibrated diode. Photodiode arrays with 50% QE, four orders of magnitude out-of-band rejection ratio, and low leakage current (<<1 nA@15 V reverse bias for 250-um pixel size) were demonstrated. Figure 11 shows measured external QE for these devices with no applied voltage; the QE increases with voltage due to voltage-enhanced collection of carriers. At higher voltages (not shown), carrier multiplication (gain) increases the measured photocurrent.

**Figure 12:**
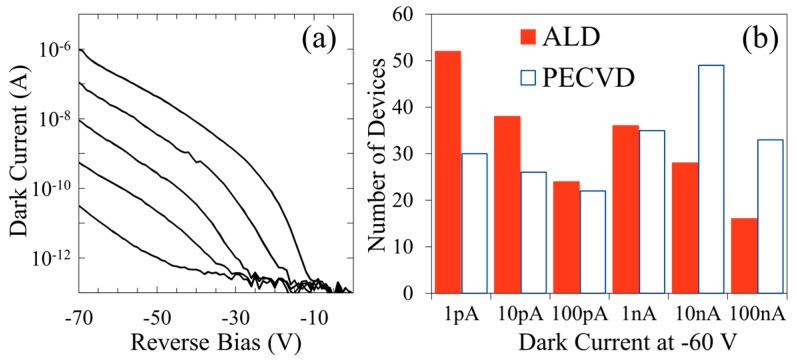
(**a**) Example of the variation in reverse bias behavior observed for ALD-passivated GaN APDs; (**b**) Histogram of ~200 devices with 25-μm diameter samples receiving a sidewall passivation layer of ALD Al_2_O_3_ or PECVD SiO_2_. First appeared in *MRS Proceedings* [50].

**Figure 13:**
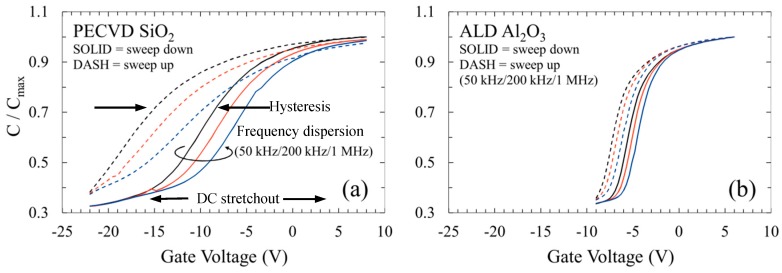
High-frequency CV characteristics for MOS capacitors fabricated on n-type GaN. A large reduction in the qualitative indicators of charge trapping—including voltage hysteresis, frequency dispersion, and stretchout—is observed for devices fabricated with (**b**) ALD Al_2_O_3_ compared to those with (**a**) PECVD SiO_2_.

The use of ALD Al_2_O_3_ as a sidewall passivation layer has resulted in the reduced occurrence of premature breakdown in mesa p-i-n GaN APDs when compared to devices fabricated with a more common plasma-enhanced chemical vapor deposition (PECVD) SiO_2_ passivation. Mesa APDs with diameters ranging from 25 to 100 µm show a significant reduction in median dark current for the ALD-passivated devices (Figure 12). The reduction in median dark current was most significant for the smallest devices, showing an order of magnitude improvement at reverse biases near avalanche. The interfacial effect of ALD Al_2_O_3_ was investigated by fabricating MOS capacitors, which show a large reduction in both slow trapping and faster interface states compared to SiO_2_-passivated devices (Figure 13).

**Figure 14:**
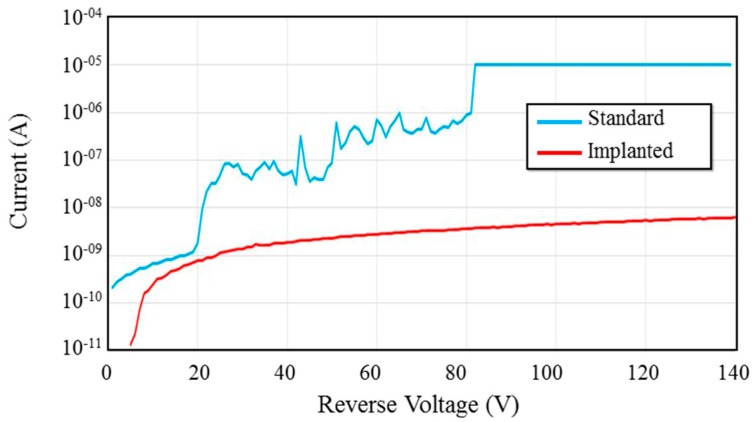
Current-voltage characteristics for an APD without contact-edge ion implantation (blue) and with implantation (red). Dark current and irreversible damage present in the unimplanted sample are reduced or eliminated by the implantation. First published in *IEEE Photonic Technology Letters* [51].

We have also shown that the high-field region around the top device contact is a significant source of avalanche dark current. One method for reducing this dark current component is ion implantation. Implantation around the contact perimeter creates high-resistance region that spreads the potential drop at the contact edge over a larger area, greatly reducing the electric field at the edges. It also creates a disordered region of heavy scattering that prevents electrons from acquiring the energy necessary for multiplication. This reduced field prevents premature breakdown at the contact edges. We have shown a large reduction in dark current and voltage-induced damage using this method (Figure 14) [51]. Innovative passivation methods using other materials can be explored to further reduce dark current in our APD devices.

**Figure 15:**
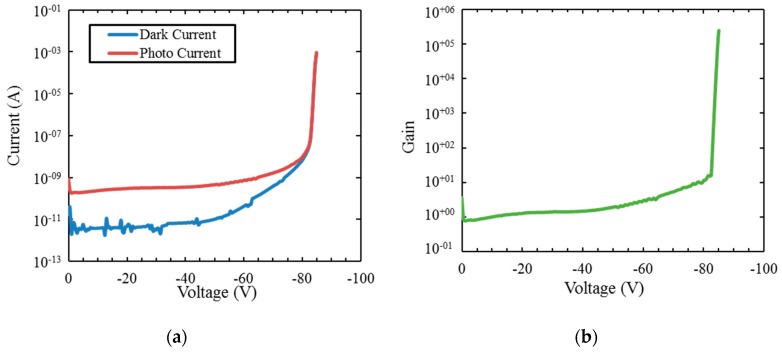
(**a**) Dark current (blue) and photocurrent (red) for a GaN p-i-n APD. (**b**) Gain derived from Figure 16a is shown in green. Avalanche gains of >10^5^ have been measured on these devices.

Avalanche operation has been demonstrated in GaN p-i-n APDs. Figure 15 shows characteristics from these devices, with large avalanche gain and low dark current. The gain values are competitive with the current state of the art for GaN APDs. These data demonstrate high-efficiency APDs resulting from our ability to grow low-defect-density III-N material.

**Figure 16:**
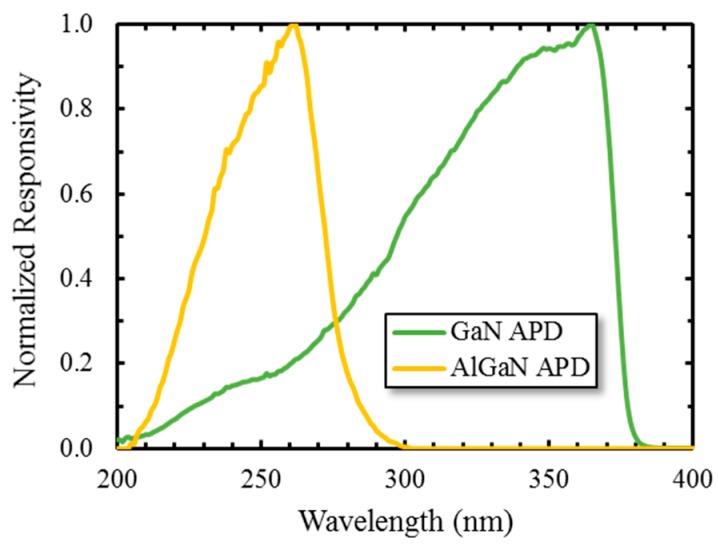
Comparison of the spectral response of GaN and Al_.4_Ga_.6_N APDs. First published in *Journal of Electronic Materials* [52].

Properties of the III-Nitride family of materials can be tuned by varying the concentration of Al in Al_x_Ga_1−x_N; capitalizing on this phenomenon we have recently demonstrated avalanche breakdown with high gain in the solar blind region. Figure 16 shows a comparison of the spectral response of GaN and Al_.4_Ga_.6_N APDs; the latter shows a detection cutoff at 280 nm in the solar blind region, a dramatic blue shift relative to the former.

**Figure 17:**
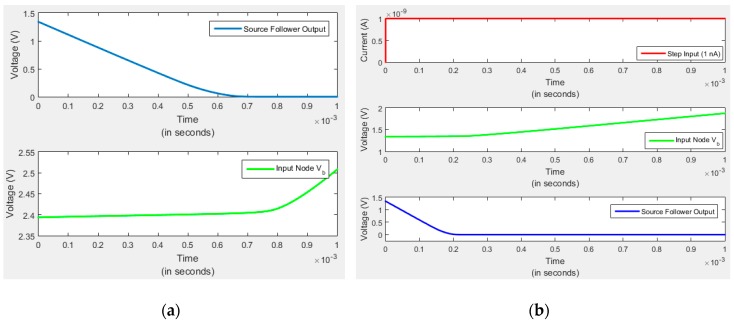
(**a**) As photodiode current starts to flow, the voltage at the node V_b_ rises from its initial value. As this occurs, the output voltage (source follower output) drops to 0. (**b**) A step response is simulated for a sudden increase in the input current from 0 to 1 nA. As soon as the input current rises to 1 nA, the output nodes settle to their steady state value without any ringing or overshoot.

As previously mentioned, work has also been done to develop a readout device for AlGaN-based APDs. Among the specialized requirements for these readouts is the ability to operate at high device bias voltages (in some cases V > 100V). Measurements from recently-fabricated readout devices have confirmed the functionality of the chip. Here we present transient and AC simulation results performed using the Cadence 6.1.5 modeling software. The transient modeling of the CTIA starts with the “reset” switch initially closed and released at t = 0. When the switch is opened, the photodiode current starts to be integrated and the voltage at the amplifier inverting input, V_b_, begins to rise, causing the amplifier output voltage, V_out1_, to fall. The small rise in V_b_ is due to the finite gain of the amplifier, with ΔV_b_ = −ΔV_out1_/A and ΔV_out1_ ≈ −Q/C_fb_, where A is the open loop gain and Q is the integrated input charge. V_out1_ drops from its initial value down to 0 V, as reflected in the output of the source follower, shown in Figure 17a). The source follower saturates somewhat sooner than the amplifier, and at this point the capacitor must be reset for another integration cycle.

We have also shown that the system is stable. Figure 17b shows a simulated step response for a sudden rise in the input current from 0 to 1 nA during which the output nodes were observed and plotted. The moment the input current rises to 1 nA, the output nodes settle to their steady state value without any ringing or overshoot.

**Figure 18:**
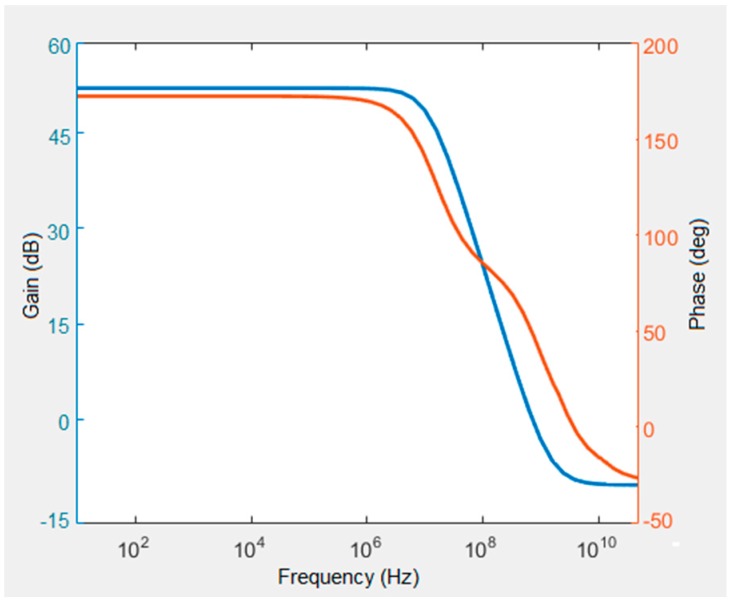
Frequency response of the readout circuit. The gain is around 45 dB with a 3 dB frequency of 10 MHz, close our target value. The modeling results indicate that the system operation is stable within the designed bandwidth.

Finally, Figure 18 shows a simulation of the open loop gain. Here the gain obtained is ~45 dB, while the 3 dB frequency is 10 MHz, close our target approximation. Furthermore, the system is stable with a phase margin of 66°.

## 4. Summary and Conclusions

Silicon-based and III-Nitride-based single photon counting ultraviolet detectors are described. In silicon, photon counting is achieved using EMCCD architecture. Although APD structure are capable of photon counting operation, the APDs used here are operated in the linear mode and are discussed because of their use to demonstrate detector-integrated visible rejection filters. EMCCDs are back illuminated and passivated using superlattice doping and delta doping. Antireflection coatings are developed using standard techniques for single and multilayerd coatings and are implemented using atomic layer deposition. Out-of-band rejection in silicon is achieved through detector-integrated metal-dielectric stacks as visible rejection filters. Avalanche photodiodes structures are grown and processed using GaN and AlGaN materials to take advantage of the wide bandgap of these materials for visible blindness. Readout is achieved by hybridization through a CMOS readout integrated circuit. A simple CTIA ROIC designed for this project has been fabricated. Both approaches show excellent promise. Silicon-based devices are ready for deployment and III-N arrays will have applications in longer-term missions. The first approach takes advantage of the maturity and mass production of silicon imaging while the second approach takes advantage of intrinsic materials characteristics. In both cases, surface and interface engineering and nano-scale materials growth and processing play key roles in achieving high quantum efficiency and low dark current. 

## Supplementary Materials

More information can be obtained regarding the techniques, equipment, and related projects described here at http://microdevices.jpl.nasa.gov/infrastructure/.

## Abbreviations

The following abbreviations are used in this manuscript:

AFM	Atomic Force Microscopy
ALD	Atomic Layer Deposition
APD	Avalanche PhotoDiode
ARC	AntiReflection Coatings
BSI	BackSide Illumination
CCD	Charge Coupled Detector
CGM	CircumGalactic Medium
CIC	Clock Induced Charge
CMOS	Complementary Metal Oxide Semiconductor
CTIA	Capacitive TransImpedance Amplifier
CV	Capacitance-Voltage
EMCCD	Electron Multiplying CCD
GALEX	GALaxy Evolution eXplorer
HST	Hubble Space Telescope
HVPE	Hydride Vapor Phase Epitaxy
IGM	InterGalactic Medium
MBE	Molecular Beam Epitaxy
MCP	MicroChannel Plate
MDF	Metal Dielectric Filter
MOCVD	Metal Organic Chemical Vapor Deposition
MOS	Metal Oxide Semiconductor
NMOS	N-type Metal Oxide Semiconductor
PECVD	Plasma Enhanced Chemical Vapor Deposition
PMOS	P-type Metal Oxide Semiconductor
PMT	PhotoMultiplier Tube
QE	Quantum Efficiency
QEH	QE Hysteresis
RMD	Radiation Monitoring Devices, Inc.
ROIC	Read Out Integrated Circuit
VLSI	Very-large-scale Integration
WF/PC	Wide Field/Planetary Camera

## References

[1] Clarke J.T., Ajello J., Ballester G.E., Ben Jaffel L., Connerney J.E.P., Gerard J.-C., Gladstone G.R., Pryor W.R., Tobiska K., Trauger J. (1999). HST/STIS images of uv auroral footprints from Io, Europa, and Ganymede. Bull. Am. Astron. Soc..

[2] McGrath M.A., Feldman P.D., Strobel D.F., Retherford K., Wolven B., Moos H.W. (2000). HST/STIS ultraviolet imaging of Europa. Bull. Am. Astron. Soc..

[3] Woodgate B.E., Kimble R.A., Bowers C.W., Kraemer S., Kaiser M.E., Grady J.F., Loiacono J.J., Brumfield M., Feinberg L.D., Gull T.R. (1998). The space telescope imaging spectrograph design. Publ. Astron. Soc. Pacific.

[4] Green J.C., Froning C.S., Osterman S., Ebbets D., Heap S.H., Leitherer C., Linsky J.L., Savage B.D., Sembach K., Michael Shull J. (2012). The cosmic origins spectrograph. Astrophys. J..

[5] Morrissey P. (2006). A GALEX instrument overview and lessons learned. Proc. SPIE.

[6] Schiminovich D., Ilbert O., Arnouts S., Milliard B., Tresse L., Le Fèvre O., Treyer M., Wyder T.K., Budavári T., Zucca E. (2005). The GALEX -VVDS measurement of the evolution of the far-ultraviolet luminosity density and the cosmic star formation rate. Astrophys. J..

[7] Feldman P.D., Steffl A.J., Parker J.W., A’Hearn M.F., Bertaux J.L., Stern S.A., Weaver H.A., Slater D.C., Versteeg M., Throop H.B. (2011). Rosetta-Alice observations of exospheric hydrogen and oxygen on Mars. Icarus.

[8] Feldman P.D., A’Hearn M.F., Bertaux J.-L., Feaga L.M., Parker J.W., Schindhelm E., Steffl A.J., Stern S.A., Weaver H.A., Sierks H. (2015). Measurements of the near-nucleus coma of comet 67P/Churyumov-Gerasimenko with the Alice far-ultraviolet spectrograph on Rosetta. Astron. Astrophys..

[9] Stern S.A., Feaga L.M., Schindhelm E., Steffl A., Parker J.W., Feldman P.D., Weaver H.A., A’Hearn M.F., Cook J., Bertaux J.-L. (2015). First extreme and far ultraviolet spectrum of a Comet Nucleus: Results from 67P/Churyumov-Gerasimenko. Icarus.

[10] Stern S.A., Scherrer J., Slater D.C., Gladstone G.R., Dirks G., Stone J., Davis M., Versteeg M., Siegmund O.H.W. (2005). ALICE: The ultraviolet imaging spectrograph aboard the New Horizons Pluto mission spacecraft. Proc. SPIE.

[11] Slater D.C., Davis M.W., Olkin C.B., Scherrer J., Stern S.A. (2005). Radiometric performance results of the New Horizons’ ALICE UV imaging spectrograph. Proc. SPIE.

[12] Esposito L.W., Barth C.A., Colwell J.E., Lawrence G.M., Mcclintock W.E., Stewart A.I.F., Keller H.U., Korth A., Lauche H., Festou M.C. (2004). The Cassini ultraviolet imaging spectrograph investigation. Space Sci. Rev..

[13] Jerram P., Pool P.J., Bell R., Burt D.J., Bowring S., Spencer S., Hazelwood M., Moody I., Catlett N., Heyes P.S. (2001). The LLCCD: Low-light imaging without the need for an intensifier. Proc. SPIE.

[14] Hynecek J. (2001). Impactron—A new solid state image intensifier. IEEE Trans. Electron. Devices.

[15] Niclass C., Favi C., Kluter T., Gersbach M., Charbon E. (2008). A 128 × 128 single-photon image sensor with column-level 10-bit time-to-digital converter array. IEEE J. Solid State Circuits.

[16] Hoenk M.E., Nikzad S., Carver A.G., Jones T.J., Hennessy J., Jewell A.D., Sgro J., Tsur S., McClish M., Farrell R. (2014). Superlattice-doped silicon detectors: Progress and prospects. Proc. SPIE.

[17] Hoenk M.E., Carver A.G., Jones T.J., Dickie M., Cheng P., Greer F., Nikzad S., Sgro J., Tsur S. The DUV stability of superlattice-doped CMOS detector arrays. Proceedings of the International Image Sensor Workshop.

[18] Nikzad S., Hoenk M.E., Carver A.G., Jones T.J., Greer F., Hamden E., Goodsall T. High Throughput, High Yield Fabrication of High Quantum Efficiency Backilluminated Photon Counting, Far UV, UV, and Visible Detector Arrays. Proceedings of the International Image Sensor Workshop.

[19] Harding L.K., Demers R.T., Hoenk M., Nemati B., Cherng M., Michaels D., Peddada P., Loc A., Bush N., Hall D. (2015). Technology Advancement of the CCD201-20 EMCCD for the WFIRST-AFTA Coronograph Instrument: Sensor characteriation and radiation damage. J. Astron. Telesc. Instrum. Syst..

[20] Hamden E.T., Lingner N., Kyne G., Morrissey P., Martin D.C. (2015). Noise and dark performance for FIREBall-2 EMCCD delta-doped CCD detector. Proc. SPIE.

[21] Reinheimer A. (2012). Personal Communication.

[22] Daigle O., Gach J.-L., Guillaume C., Carignan C., Balard P., Boisin O. (2004). L3CCD results in pure photon-counting mode. Proc. SPIE.

[23] Daigle O., Djazovski O., Laurin D., Doyon R., Artigau É. (2012). Characterization results of EMCCDs for extreme low-light imaging. Proc. SPIE.

[24] Shortes S.R., Chan W.W., Rhines W.C., Barton J.B., Collines D.R. (1974). Characteristics of thinned backside-illuminated charge-coupled device imagers. Appl. Phys. Lett..

[25] Stoller A., Speers R., Opresko S. (1970). A new technique for etch thinning silicon wafers. RCA Rev..

[26] Kern W. (1978). Chemical etching of silicon, germanium, gallium arsenide, and gallium phosphide. RCA Rev..

[27] Collins N.R., Boehm N., Delo G., Foltz R.D., Hill R.J., Kan E., Kimble R.A., Malumuth E., Rosenberry R., Waczynski A. (2009). Wide field camera 3 CCD quantum efficiency hysteresis: Characterization and mitigation. Proc. SPIE.

[28] Hoenk M.E., Grunthaner P.J., Grunthaner F.J., Terhune R.W., Fattahi M., Tseng H.-F. (1992). Growth of a delta-doped silicon layer by molecular beam epitaxy on a charge-coupled device for reflection-limited ultraviolet quantum efficiency. Appl. Phys. Lett..

[29] Hoenk M.E., Grunthaner P.J., Grunthaner F.J., Terhune R.W., Fattahi M.M. (1992). Epitaxial Growth of p+ Silicon on a Backside-thinned CCD for Enhanced UV Response. Proc. SPIE.

[30] Hoenk M.E., Carver A.G., Jones T.J., Dickie M.R., Sgro J., Tsur S. Superlattice-doped imaging detectors: Structure, physics and performance. Proceedings of the Scientific Detectors Workshop.

[31] Nikzad S., Hoenk M.E., Grunthaner P.J., Terhune R.W., Grunthaner F.J., Winzenread R., Fattahi M., Tseng H.-F., Lesser M. (1994). Delta-doped CCDs: High QE with long-term stability at UV and visible wavelengths. Proc. SPIE.

[32] Nikzad S., Jones T.J., Elliott S.T., Cunningham T.J., Deelman P.W., Walker A.B.C., Oluseyi H.M. (2000). Ultrastable and uniform EUV and UV detectors. Proc. SPIE.

[33] Hoenk M.E., Jones T.J., Dickie M.R., Greer F., Cunningham T.J., Blazejewski E.R., Nikzad S. (2009). Delta-doped back-illuminated CMOS imaging arrays: Progress and prospects. Proc. SPIE.

[34] Nikzad S., Hoenk M.E., Greer F., Jacquot B., Monacos S., Jones T.J., Blacksberg J., Hamden E., Schiminovich D., Martin D.C. (2012). Delta doped electron multiplies CCD with absolute quantum efficiency over 50% in the near to far ultraviolet range for single photon counting applications. Appl. Opt..

[35] Greer F., Hamden E., Jacquot B.C., Hoenk M.E., Jones T.J., Dickie M.R., Monacos S.P., Nikzad S. (2013). Atomically precise surface engineering of silicon CCDs for enhanced UV quantum efficiency. J. Vac. Sci. Technol. A.

[36] Jewell A.D., Hennessy J., Hoenk M.E., Nikzad S. (2013). Wide band antireflection coatings deposited by atomic layer deposition. Proc. SPIE.

[37] Jewell A.D., Hamden E.T., Ong H.R., Hennessy J., Goodsall T., Shapiro C., Cheng S., Carver A., Hoenk M., Schiminovich D. (2015). Detector performance for the FIREBall-2 UV experiment. Proc. SPIE.

[38] Hoenk M.E. (2013). Surface Passivation by Quantum Exclusion Using Multiple Layers. U.S. Patent.

[39] Hamden E.T., Greer F., Hoenk M.E., Blacksberg J., Dickie M.R., Nikzad S., Martin D.C., Schiminovich D. (2011). Ultraviolet antireflection coatings for use in silicon detector design. Appl. Opt..

[40] Hamden E.T., Jewell A.D., Shapiro C.A., Cheng S.R., Goodsall T.M., Hennessy J., Nikzad S., Hoenk M.E., Jones T.J., Gordon S. CCD detectors with greater than 80% QE at UV wavelengths.

[41] Bates B., Bradley D.J. (1966). Interference filters for the far ultraviolet (1700 A to 2400 A). Appl. Opt..

[42] Scalora M., Bloemer M.J., Pethel A.S., Dowling J.P., Bowden C.M., Manka A.S. (1998). Transparent, metallo-dielectric, one-dimensional, photonic band-gap structures. J. Appl. Phys..

[43] Renk K.F., Genzel L. (1962). Interference filters and Fabry-Perot interferometers for the far infrared. Appl. Opt..

[44] Sigalas M.M., Chan C.T., Ho K.M., Soukoulis C.M. (1995). Metallic photonic band-gap materials. Phys. Rev. B.

[45] Piegari A., Bulir J. (2006). Variable narrowband transmission filters with a wide rejection band for spectrometry. Appl. Opt..

[46] Bloemer M.J., Scalora M. (1998). Transmissive properties of Ag/MgF_2_ photonic band gaps. Appl. Phys. Lett..

[47] Hennessy J., Jewell A.D., Hoenk M.E., Nikzad S. (2015). Metal-dielectric filters for solar-blind silicon ultraviolet detectors. Appl. Opt..

[48] Blacksberg J., Hoenk M.E., Elliott S.T., Holland S.E., Nikzad S. (2005). Enhanced quantum efficiency of high-purity silicon imaging detectors by ultralow temperature surface modification using Sb doping. Appl. Phys. Lett..

[49] Blacksberg J., Nikzad S., Hoenk M.E., Holland S.E., Kolbe W.F. (2008). Near-100% quantum efficiency of delta doped large-format UV-NIR silicon imagers. IEEE Trans. Electron Devices.

[50] Hennessy J., Bell L.D., Nikzad S., Suvarna P., Leathersich J.M., Marini J., Shahedipour-Sandvik F.S. (2014). Atomic-layer Deposition for Improved Performance of III-N Avalanche Photodiodes. MRS Online Proceeding Library.

[51] Suvarna P., Bulmer J., Leathersich J.M., Marini J., Mahaboob I., Hennessy J., Bell L.D., Nikzad S., Shahedipour-Sandvik F. (2015). Ion implantation-based edge termination to improve III-N APD reliability and performance. IEEE Photonics Technol. Lett..

[52] Suvarna P., Tungare M., Leathersich J.M., Agnihotri P., Shahedipour-Sandvik F., Bell L.D., Nikzad S. (2013). Design and growth of visible-blind and solar-blind III-N APDs on sapphire substrates. J. Electron. Mater..

